# Use of Internet Audience Measurement Data to Gauge Market Share for Online Health Information Services

**DOI:** 10.2196/jmir.7.3.e31

**Published:** 2005-07-01

**Authors:** Fred B Wood, Dennis Benson, Eve-Marie LaCroix, Elliot R Siegel, Susan Fariss

**Affiliations:** ^1^National Library of MedicineNational Institutes of HealthBethesda, MDUSA

**Keywords:** Internet, World Wide Web, information services, information dissemination, audience research, evaluation studies

## Abstract

**Background:**

The transition to a largely Internet and Web-based environment for dissemination of health information has changed the health information landscape and the framework for evaluation of such activities. A multidimensional evaluative approach is needed.

**Objective:**

This paper discusses one important dimension of Web evaluation—usage data. In particular, we discuss the collection and analysis of external data on website usage in order to develop a better understanding of the health information (and related US government information) market space, and to estimate the market share or relative levels of usage for National Library of Medicine (NLM) and National Institutes of Health (NIH) websites compared to other health information providers.

**Methods:**

The primary method presented is Internet audience measurement based on Web usage by external panels of users and assembled by private vendors—in this case, comScore. A secondary method discussed is Web usage based on Web log software data. The principle metrics for both methods are unique visitors and total pages downloaded per month.

**Results:**

NLM websites (primarily MedlinePlus and PubMed) account for 55% to 80% of total NIH website usage depending on the metric used. In turn, NIH.gov top-level domain usage (inclusive of NLM) ranks second only behind WebMD in the US domestic home health information market and ranks first on a global basis. NIH.gov consistently ranks among the top three or four US government top-level domains based on global Web usage. On a site-specific basis, the top health information websites in terms of global usage appear to be WebMD, MSN Health, PubMed, Yahoo! Health, AOL Health, and MedlinePlus. Based on MedlinePlus Web log data and external Internet audience measurement data, the three most heavily used cancer-centric websites appear to be www.cancer.gov (National Cancer Institute), www.cancer.org (American Cancer Society), and www.breastcancer.org (non-profit organization).

**Conclusions:**

Internet audience measurement has proven useful to NLM, with significant advantages compared to sole reliance on usage data from Web log software. Internet audience data has helped NLM better understand the relative usage of NLM and NIH websites in the intersection of the health information and US government information market sectors, which is the primary market intersector for NLM and NIH. However important, Web usage is only one dimension of a complete Web evaluation framework, and other primary research methods, such as online user surveys, usability tests, and focus groups, are also important for comprehensive evaluation that includes qualitative elements, such as user satisfaction and user friendliness, as well as quantitative indicators of website usage.

## Introduction

### Shift to the Internet

The advent of the Internet and World Wide Web has fundamentally changed the competitive environment for health information services of all kinds. Over the last decade, the National Library of Medicine (NLM), like many others, has made a major transition to Internet and Web-based dissemination of health information. Dissemination of information via varied websites is by far the dominant channel used by NLM and by most other units of the National Institutes of Health (NIH) for making health information available to consumers, health professionals, librarians, and researchers.

The shift to the Internet has necessitated a rethinking and transition in information dissemination evaluation methods as well. In the pre-Internet days, NLM relied heavily on user surveys targeted to identifiable users who were known to NLM because of the requirement to register. Now, in the Web environment, most NLM (and other NIH) Web-based information services do not require registration; indeed, NLM in particular emphasizes the protection of user privacy and does not collect, as a matter of routine, any identifiable information about its users. The only exceptions are specialized services such as email updates or stored searches where users, of their own volition, provide contact information in order to receive these services.

This situation has vastly compounded the difficulty of not only getting feedback from users, but of understanding and tracking the relative market positions of health information providers. To a significant degree, all health information providers face similar challenges. However, federal government providers, such as NLM and other NIH units, are further limited because of the prohibitions on the use of persistent cookies, behavioral tracking, and personal identifiers—restrictions that do not apply to many private sector health information providers.

### Multidimensional Approach

In order to address the challenges of evaluating Web-based information dissemination, NLM has developed a multidimensional approach to Web evaluation [1] (also see [2,3]). This approach includes the following dimensions: usability testing (heuristic or expert review, usability lab testing, informal usability feedback); user feedback (online user survey, online survey of external panel, online or face-to-face focus group, nationwide syndicated survey, unsolicited user feedback); usage data (Web log data analysis, Internet audience measurement); Web/Internet performance (page download times, latency and traceroute, throughput); and special outreach projects.

This approach is intended in part to better understand NLM's position in the health information arena. The focus of this paper is NLM's use of Internet audience measurements services as a primary methodology to estimate NLM's and NIH's share of the health information market. To a large degree, Internet audience measurement services offer the only viable means to obtain market-wide usage data, since Web log data from other information providers typically are not available. This is the case in both the public and private sectors.

Additionally, Web log data definitions are highly variable from site to site and depend on site- and software-specific details. This can limit the validity (and utility) of log data for comparative purposes. Internet audience measurement services offer the possibility of applying the same methodology and definitions across the board, for all websites and subsites being measured and compared.

In this paper, we apply the Internet audience measurement methodology to develop estimates of NLM's and NIH's positions in the health information (and also federal government) market sectors. We also examine, as a case study, both MedlinePlus Web log data and external Internet audience measurement data on the most heavily used cancer-specific health information websites.

## Methods

### Measuring Internet Audience

NLM realized early in its transition to Internet and Web-based information dissemination that new audience measurement methods would be needed. NLM now has several years' experience with various Internet audience measurement services and currently subscribes to two commercial services—comScore [4] and Nielsen/NetRatings [5]. comScore had its early roots in the now defunct PCData measurement service, which was transformed in 2001 by comScore into the now defunct netScore measurement service, which was further transformed in 2003 by integrating it with the MediaMetrix service. Nielsen/NetRatings is a wholly owned subsidiary of AC Nielsen, well known for the use of panels for measuring television audience shares. NetRatings began independently and was acquired by AC Nielsen in the 1990s.

Both services use a similar overall approach in that estimates of overall website usage are developed based on actual Web usage by panels of users. The two services vary in their approaches to panel recruitment, size and scope of panels, estimating algorithms, projection methodologies, and geographic and sectoral coverage. At the core, however, both use panels of Web users who volunteer to have their Web usage monitored 24 hours a day, 7 days a week. The raw Web usage data are then adjusted using US census data and other survey data to produce estimates extrapolated to the defined market sector. The specific estimating algorithms used by each company are considered proprietary and differ considerably.

### Comparing the Services

Nielsen/NetRatings uses a panel of about 60000 persons in the United States, and it prepares estimated usage levels for the US home and office markets and total US market. Nielsen/NetRatings has affiliates in select other countries but does not as yet prepare integrated global usage estimates. comScore uses a panel of about 1.2 million persons in the United States augmented by about 300000 persons in other countries. comScore provides usage estimates for the US home, office, and school markets and total US market plus the non-US usage, which together provide global usage estimates.

Both services use similar metrics. The primary metrics used by NLM for Internet audience measurement, whether based on external panels or internal Web log software, are the following: unique visitors (number of different users); total visitors (total number of users including repeat visitors); total visits (number of times the NLM sites are visited in a given period); and pages downloaded (total number of Web pages downloaded by all users).

For this paper, we have used only Internet audience measurement data from comScore because it is the only one of the two vendors that provides estimated worldwide website usage. This is particularly important in the case of NLM, since half or more of NLM's Web usage originates from countries other than the United States. Also, worldwide usage data are the only data that can reasonably be compared with Web log data. Also, in this paper, we use only the comScore data based on measurement of traffic from the computers of participating panelists. These are known as machine-based panel data and are more comparable to the data collected by Web log software. Machine-based panel data could undercount the total number of users due to multiple persons using the same computer. All data in this paper are machine-based—either from the comScore panel or from NLM's Web log software—in order to help assure comparability to the extent possible.

## Results

### Cross-Validation with Web Log Data

NLM has made efforts to cross-validate Internet audience measurement data from external panels against internal Web log data. Only comScore data can be used for this type of cross-correlation, since the Web log data measure global usage. As noted, the comScore methodology measures Web usage of panel members and then extrapolates to US and global estimates based on demographic factors and assumptions. In comparison, Web log software measures the number of IP addresses that visitors are using, not the users directly, and includes IP addresses from any locale worldwide. The assumption is that the number of different IP addresses measured in a given period of time roughly correlates with the number of actual users. But single users with dynamic IP addresses and multiple users with the same IP address are two examples of ways in which the IP data could be misleading. Thus, both methods are subject to varied sources of error, and precise correlations between Web log data and external Internet audience data would not be expected.

Table 1 shows comparisons between comScore global usage data and Web log data for the two most heavily used NLM websites—PubMed and MedlinePlus—for three metrics, in September 2003. Table 2 shows the same information for October 2004.

**Table 1:** Comparison of comScore and Web log data for PubMed and MedlinePlus, September 2003

		**UniqueVisitors(millions)**	**TotalVisits(millions)**	**TotalPages Downloaded(millions)**
PubMed				
	Web log^*^	4.2	7.6^†^	193
	comScore	3.8	10.2	62
MedlinePlus				
	Web log^‡^	2.7	5.6	23
	comScore	3.4	5.2	20

^*^ Data are from custom Web log software installed on PubMed server.

^†^ Estimated

^‡^ Data are from WebTrends Web log software installed on MedlinePlus server.

**Table 2:** Comparison of comScore and Web log data for PubMed and MedlinePlus, October 2004

		**UniqueVisitors(millions)**	**TotalVisits(millions)**	**TotalPages Downloaded(millions)**
PubMed				
	Web log^*^	10	21^†^	235
	comScore	5.7	12.8	117
MedlinePlus				
	Web log^‡^	6.2	10.5	59
	comScore	5.6	8.7	30

^*^ Data are from custom Web log software installed on PubMed server.

^†^ Estimated

^‡^ Data are from WebTrends Web log software installed on MedlinePlus server.

The usage data compare very well for MedlinePlus across all metrics—unique visitors, total visits, and total pages downloaded—in the first sample month (September 2003) and for unique visitors and total visits in the second sample month (October 2004), but not total pages downloaded. The comScore data appear to undercount the number of MedlinePlus pages downloaded in October 2004, which may reflect differences in definitions of what data are captured and in usage patterns of the comScore panelists. For example, tutorials are one of the most popular MedlinePlus features, and the Web log software equates each tutorial to many pages downloaded (each sequential view of the tutorial is counted as a separate page). It is unclear to what extent the comScore methodology captures tutorial use and other MedlinePlus special features, such as the link-outs, on a basis directly comparable to Web log software.

For PubMed, the September 2003 data compare very well for unique visitors, fairly well for total visits, and not very well for total pages downloaded; the October 2004 data suggest that comScore is undercounting PubMed usage across all three metrics by 50% to 75%. The PubMed discrepancies are probably due in part to the under-representation of researchers and scientists on the comScore panel, as well as to differences in definitions of what is considered a “page viewed” with Web log data versus comScore data. Researchers and scientists are a core PubMed user group and likely are very intensive users. This would translate into a heavy volume of visits and pages downloaded; thus, if they are under represented on the panel, this would result in lower than expected usage data. While the comScore panel gives special attention to the college sector, the emphasis is primarily on students, not on faculty and research scientists who would likely be the more intensive users of PubMed. Overall, the apparent correlation between comScore and Web log data was judged to be good in September 2003 but mixed in October 2004 due to the PubMed undercounting. The latter may be exacerbated in recent months because Google has indexed PubMed, which appears to have significantly further increased the number of site visitors.

### NLM as a Percentage of NIH Web Usage

One of the goals of NLM's use of Internet audience measurement data is to better understand NLM's position within the broader NIH Internet and Web usage environment. This is important to know because the Internet audience measurement services generally collect data by top-level domain, such as NIH.gov. This makes it difficult to track subdomain usage unless the subdomain (or group of subdomains) represents a large part of the top-level domain usage.

Accordingly, NLM has requested special drill-down data from both vendors in order to be able to separate usage of NLM websites from usage of other NIH websites. Table 3 shows the estimated percentages of total NIH Web usage that are attributable to NLM's National Center for Biotechnology Information (NCBI), including, predominately, PubMed, MedlinePlus, and other NLM websites combined.

**Table 3:** NLM website usage as a percentage of NIH usage, October 2004, based on comScore data

	**Unique Visitors(Reach)(%)**	**Total Pages(Share)(%)**
NCBI (including PubMed)	45	64+
MedlinePlus	39	14
Other NLM websites	4	1
Totals	55-75 (estimated; not additive)	80+ (additive)

Unique visitors are not strictly additive since users can visit more than one NIH website in a given month. However, the usage data suggest that an estimated 55% to 70% of the total unique visitors per month to all NIH websites are accounted for by usage of NLM websites. Total pages downloaded per month are additive, and these data highlight the large percentage of pages downloaded that is attributed to users of NLM websites—about 80% (or more, if the comScore data undercount PubMed pages downloaded).

Overall, these results suggest that, at least as a rough approximation, NIH top-level domain usage data can be used as a reasonable surrogate of NLM website usage, since NLM website usage accounts for such a large part of overall NIH website usage.

### US Home Health Information Space

The next step in the analysis was to look at the US home health information space with regard to the leading general purpose health information sites. (Specialized websites such as ediets.com were excluded for this purpose.) Using US home data from comScore, the top five websites in terms of monthly unique visitors for September 2004 were the following:

- WebMD: 2.5 million
- NIH.gov: 2.4 million (top-level domain inclusive of NLM)
- AOL Health (powered by WebMD): 1.7 million
- Yahoo! Health: 1.2 million
- MSN Health (at the time, powered by WebMD): 950000

Thus in the US home market, WebMD and NIH.gov were virtually tied in usage, although WebMD would be the clear leader if credited with the usage on AOL Health and MSN Health that both use WebMD for their consumer health information portals.

The second tier of health information websites in the US home market includes the following (numbers are unique visitors per month):

- CDC.gov (Centers for Disease Control, US Department of Health and Human Services): 740000
- MayoClinic.com: 427000
- KidsHealth.org (Nemours Foundation): 448000
- Medscape.com (geared to physicians and other health professionals; now part of WebMD): 263000
- FamilyDoctor.org (American Academy of Family Physicians): 243000
- HHS.gov (the main DHHS website): 384000
- AMA-assn.org (American Medical Association): 174000
- Intelihealth.com (with Harvard Medical School): 120000

Taken as a whole, it would appear the WebMD and NIH.gov are the clear leaders in the US home health information market based on unique visitors per month.

### Global Health Information Space

Over the last several years, NLM has detected an increase in non-US usage of NLM websites. This trend was confirmed by Internet audience measurement data. The global increase has been significant enough to keep NIH.gov overall Web usage in the number one position in the global health information space, with WebMD.com close behind in number two (Table 4), as measured by unique visitors per month. In terms of total pages downloaded per month, NIH.gov Web usage maintained a significant edge over WebMD (due mainly to large page downloads by PubMed users). comScore believes that most of the increase in WebMD usage can be attributed to an increase in advertising and promotion of the WebMD brand combined with WebMD's strategic partnerships and acquisitions.

**Table 4:** NIH.gov versus WebMD usage in the global health information space, based on comScore data

		**Sept 2002**	**Sept 2003**	**Sept 2004**
**Unique Visitors per Month (millions)**				
	NIH.gov	6.6	9.8	12.3
	WebMD	5.3	5.9	11.1
**Total Pages Downloaded per Month (millions)**				
	NIH.gov	87.7	114.8	184.0
	WebMD	64.6	81	107.8

**Table 5:** Illustrative worldwide leading health information websites, April 2004 and April 2005, based on comScore data

**Website**	**Unique Visitors in April 2004** **(millions)**	**UniqueVisitors in April 2005** **(millions)**
NIH.gov (all websites)	11.3	17.2
NLM websites (subset of NIH.gov)	7.6	12.1
WebMD (main corporate website)	7.5	15.5
AOL Health^*^	4.9	6.1
PubMed (subset of NLM)	3.8	7.4
Yahoo Health	5.0	6.5
MedlinePlus (subset of NLM)	4.1	5.4
MSN Health^†^	3.7	10.1
iVillage Health	2.1	1.5
KidsHealth.org	1.5	2.9
Medscape.com	1.7	1.8
MayoClinic.com	1.1	2.0
FamilyDoctor.org	.465	1.4
AMA-assn.org	.928	1.3
Cancer.org	.690	1.1
Cancer.gov	.652	.704
Intelihealth sites	.545	.597
Breastcancer.org	.168	.327

^*^ AOL Health is powered by WebMD.

^†^ MSN health was powered by WebMD in 2004 but not in 2005.

As shown in Table 5, as of April 2005, measured usage of the NIH family of websites still exceeded that of WebMD. However, in terms of individual websites, WebMD still was the number one single website, and it exceeded the combined usage of NLM's (and NIH's) two most heavily used websites—PubMed and MedlinePlus. Further, if AOL Health was counted as part of the WebMD network (on the argument AOL uses the WebMD health portal), then the WebMD network of websites would exceed the NIH family of websites as measured by unique visitors per month. Note that MSN Health used to include the WebMD health portal, but in 2005, it changed its partnership such that WebMD still provides the content but without using the WebMD brand on MSN. Thus it can be argued that MSN Health is no longer part of the WebMD family from the perspective of Internet audience measurement of branded websites.

### Global Cancer Information Space

The two primary types of websites for disease-specific health information are general purpose health sites that include information on a wide range of diseases and conditions, and specialty websites focused on a single disease or condition.

MedlinePlus is an example of a general purpose health information portal website. For purposes of this paper, we focused on cancer information. Three major types of cancer information available through MedlinePlus are (1) individual downloadable Web pages that include cancer-related content, (2) interactive tutorials that address cancer-related topics, and (3) link-outs (known as “redirects”) from MedlinePlus to other websites that, in turn, have cancer-related content.

MedlinePlus Web log data for October 2004 indicate that about 4% of the top 360 pages downloaded are pages with cancer-related information. The topics covered included the following types of cancer: breast, prostate, skin, cervical, lung, ovarian, stomach, lymphoma, colon, bone, Hodkgin's lymphoma, colorectal, and throat or larynx. The October 2004 data show that about 16% of the tutorial usage related to cancer and covered the following topics in one way or another: prostate cancer, chemotherapy, colon cancer, mammography, brain cancer, breast lump biopsy, breast cancer, ovarian cancer, radiation therapy, skin cancer, early screening and cancer prevention, lung cancer, and melanoma.

The October 2004 Web log data also show that about 4% of all MedlinePlus link-outs (users linking or clicking out from MedlinePlus to an external website) clearly were to cancer-related websites. Three sites accounted for most of these link-outs: www.cancer.gov (National Cancer Institute), www.cancer.org (American Cancer Society), and www.breastcancer.org (maintained by an independent non-profit organization). The topics covered in these link-outs included the following: actinic keratosis, Hodgkin's disease, stomach cancer, skin cancer, prostate cancer, cervical cancer, ovarian cancer, myeloma, bone cancer, non-Hodgkin's lymphoma, stomach cancer, uterine cancer, gallbladder cancer, breast cancer, kidney cancer, liver cancer, pancreatic cancer, thyroid cancer, and endometrial cancer. It would be expected that other general purpose health websites, such as MayoClinic and WebMD, would also have a significant percentage of cancer-related page downloads and website link-outs.

Another way of looking at the data is by NIH institutes and centers. Among all the NIH units with websites, the Web log data show that, in October 2004, the second largest percentage of MedlinePlus link-outs went to the National Cancer Institute (about 4% of all link-outs).

With regard to cancer-specific websites, a review of the comScore worldwide data for August 2004 indicated that the top three sites were www.cancer.gov, www.cancer.gov, and www.breastcancer.org. Thus the global data based on the comScore external panel and the MedlinePlus Web log data appear to be consistent in identifying the most heavily used cancer-specific websites. However, it should be emphasized that there no doubt are other useful cancer-specific websites but with lower usage levels that are below the minimum cutoffs for monitoring by comScore.

### Global US Government Information Space

NLM and NIH are responsible for websites that operate in both the health information space and the US government information space. Table 6 illustrates the impact of global Web usage on the relative rankings of the top eight US government websites or top-level domains. For the months of September 2003 and September 2004, NIH.gov ranked consistently in the top three US government websites in global unique visitors and in the top four in global total pages downloaded. NOAA, USPS, and NASA, along with NIH, are in the leading group, joined by the IRS and US Department of Education during peak tax and student financial aid seasons, respectively.

Table 7 shows comparisons between the NIH.gov top-level domain and other well-known US government top-level domains. NIH.gov had several times the number of unique visitors per month than any of the other domains listed. By comparison, NIH.gov had roughly six times the number of visitors as WhiteHouse.gov and about the same number of visitors as the combined total of the WhiteHouse.gov, Army.mil, Navy.mil, AF.mil (Air Force), and CIA.gov. Using the ratios discussed earlier, the NLM subdomain usage would be roughly equivalent to, for example, the combined total of the WhiteHouse.gov, House.gov, Senate.gov, and CIA.gov (or other equivalent combinations).

**Table 6:** Relative global rankings of the top eight US government websites or top-level domains, based on comScore data

**Web Domain**	**September 2003**				**September 2004**			
	**US UniqueVisitors**	**Global UniqueVisitors**	**US TotalPages**	**Global TotalPages**	**US UniqueVisitors**	**Global UniqueVisitors**	**US TotalPages**	**Global TotalPages**
NIH.gov	3	1	4	1	3 —	2 ↓	4 —	3 ↓
NOAA.gov	2	2	2	3	1 ↑	1 ↑	1 ↑	1 ↑
USPS.com	1	3	1	2	2 ↓	3 —	2 ↓	2 —
NASA.gov	6	4	6	5	6 —	4 —	5 ↑	5 —
LOC.gov	8	5	8	7	8 —	8 ↓	8 —	8 ↓
ED.gov	4	6	3	4	4 —	5 ↑	3 —	4 —
CDC.gov	7	7	7	8	7 —	6 ↑	7 —	7 ↑
IRS.gov	5	8	5	6	5 —	7 ↑	6 ↓	6 —

Note: Arrows indicate direction of change (increase or decrease) in relative ranking (— denotes no change in ranking).

**Table 7:** NIH.gov Worldwide usage compared with other US government top-level domains, April 2005, based on comScore data

**Web Domain**	**Unique Visitors(millions)**	**Total PagesDownloaded(millions)**
NIH.gov	16.8	287.3
Army.mil	4.2	162.6
Navy.mil	3.0	43.1
CIA.gov	2.9	12.2
AF.mil (Air Force)	2.0	47.2
WhiteHouse.gov	2.3	24.8
House.gov	1.5	7.5
Senate.gov	.978	6.2

## Discussion

### Overall Value

The use of Internet audience measurement services based on external panels of Web users has proven invaluable to NLM. This method is the only known means by which NLM can understand where NLM (and NIH) websites fit into the health information and government information sectors.

The comScore Internet audience data indicate that NLM websites collectively account for the majority of NIH website usage, and that, in turn, NIH websites collectively are the most heavily used among all US government health agencies. Overall, in the global health information space, based on April 2005 data, the top six websites in terms of usage on a single site basis appear to be WebMD, MSN Health, PubMed, Yahoo! Health, AOL Health, and MedlinePlus. Given the uncertainties in the extrapolated usage data, it could be argued that these websites are in the same ball park in overall usage. On a corporate basis, NIH.gov (all websites, but powered in large part by PubMed and MedlinePlus) would appear to have somewhat greater usage than WebMD. However, WebMD would have greater usage if the traffic of its strategic partner AOL Health was included.

There is no perfect methodology for estimating website usage. Interpretation and use of such usage data should take into account the inherent limitations of the data collection and extrapolation methodology, whether it is the use of external Internet audience panels or Web log software.

### Relative Advantages

Many websites, and most major websites such as NLM's, have Web log software installed on the Web servers. The Web log software captures data on website usage, including total number of visits and visitors, unique visitors, pages downloaded, and various other usage metrics. The Web log data provide reasonable estimates of usage of the website on which the software is installed. The Web log data are subject to some error factors since the data are based on IP addresses using the website, which are used as a surrogate of the actual human users. Complications arise for users with dynamic IP addresses since a single user (especially a dial-up user) might have a new IP address assigned at each log-in. This could artificially inflate the number of unique users in a given month. On the other hand, users at universities or companies may be undercounted since these institutions often use proxy servers, which Web logs record as a single IP address. Thus, multiple users would be counted as only one user since the IP address remains the same for each user. Proxy servers can also cache downloaded pages thus also undercounting the number of pages downloaded.

Internet audience measurement data based on Web usage by external panels offer a complementary method for estimating usage, even of one's own website. Of course, the external panel methodology is itself subject to some error factors, such as variable methods of panel recruitment, selection bias in populating the panel, and uncertainties in extrapolating from panel usage to sectoral, national, or even global usage estimates. On the other hand, Web robots, agents, and crawlers may impact and possibly distort the Web log data, but not the panel data. NLM has found that the global Web usage estimates do correlate reasonably well (typically +/- 10% to 15%) for MedlinePlus when compared with Web log usage data for comparable time periods. However, it now appears that the comScore data undercount PubMed usage by 50% to 75% or more. It remains to be seen in coming months whether this difference is due to the Google indexing, under-representation on the comScore of some primary PubMed users groups (eg, biomedical scientists), or to some other factors as yet unidentified. Only global usage estimates can be compared since it is very difficult to parse out IP-based usage data for specific geographical areas or user sectors.

### Market Aggregation and Drill Down

In addition to augmenting Web log data, Internet audience measurement data based on external panels has the decided advantage of being able to provide usage estimates for other websites. Further, the data can be aggregated into market segments, and provide the basis for estimating market shares for specific websites of interest. In theory, such market share estimates could be constructed from Web log data. But as a practical matter, Web log data are considered by many organizations to be proprietary, and such data are very difficult to access by anyone outside of a website's own organization.

Based on NLM's own experience with using external Internet audience measurement data, it would appear that such data can be used to paint a reasonably accurate picture of the relevant market sectors—in NLM's case, the health information sector and US government information sector. NLM's websites are situated at the intersection of these two market sectors, or spaces.

NLM also has found the Internet audience measurement data useful for better understanding the usage distribution within a top-level domain, in this case, NIH.gov. NIH is a very large organization with over 125 websites managed by several dozens of separate organizational units. It has proven difficult to collect Web log data across so many websites. Thus, Internet audience measurement data are a more viable way to at least estimate relative usage of websites within the NIH.gov top-level domain. In earlier years, NLM requested so-called subdomain drill down data from the vendors as a custom service. Today, however, subdomain data are available, at least for websites with adequate traffic, as part of routine online data reporting.

NLM's efforts to understand and track Web usage are somewhat easier because two of NLM's websites, MedlinePlus and PubMed, appear to be the two most heavily used US government health-related websites. Together they account for between half and four-fifths of all NIH Web usage, depending on the metric used.

The dominance of the usage data by MedlinePlus and PubMed in the public sector, and by WebMD in the private sector, should not, however, obscure the importance of many health-related websites with lesser usage levels. At NLM and NIH, there are many so-called niche-market websites that focus on health information related to a specific disease, condition, or research or application area. The usage of these websites can also be tracked with Internet audience measurement data, at least for websites with 50000 to 100000 monthly unique visitors. Below that level, the panel usage data are usually too limited to assign statistical significance.

The cancer information case study illustrated how both Web log data and Internet audience data can improve understanding of usage of disease-specific health information websites.

### Conclusion

In summary, overall, Internet audience measurement data based on Web usage of external panels have proven to be quite useful to NLM. These data have allowed NLM to better understand the overall health information market space and the positioning of NLM websites within that market. The Internet audience data also lend themselves to various types of demographic and geographic analyses, which NLM intends to compare with other types of usage data, such as Web log data, and with the results of user surveys.

The external measurement approach is an important tool in NLM's arsenal of Web evaluation methods. It must be kept in mind, however, that Web usage statistics such as unique visitors and pages downloaded per month, while important, do not address the perceived quality, usefulness, or user friendliness of the referenced websites. For these key dimensions of website performance, other Web evaluation methods are needed. These include, in particular, surveys of Web users. NLM has made extensive use of online surveys of NLM website users, while these users are on the website. As a complement, members of external panels, including the Internet audience measurement panels, could be surveyed as well. We end where we began, with an emphasis on the need for a multidimensional approach to Web evaluation, of which Internet audience measurement is one of several important methods.

## Abbreviations

IP: Internet protocol
NCBI: National Center for Biotechnology Information
NIH: National Institutes of Health
NLM: National Library of Medicine
